# Decline in the seroprevalence of syphilis markers among first-time blood donors in Libreville (Gabon) between 2004 and 2016

**DOI:** 10.1186/s12889-019-6489-7

**Published:** 2019-02-08

**Authors:** Cyrille Bisseye, Jean-Marie Eko Mba, Jophrette Mirelle Ntsame Ndong, Heidi E. Kosiorek, Richard J. Butterfield, Landry Erik Mombo, Bertrand M’batchi, Mitesh J. Borad, Bolni Marius Nagalo, Jean-Pierre Allain

**Affiliations:** 1 Laboratoire de Biologie Moléculaire et Cellulaire (LABMC), Université des Sciences et Techniques de Masuku (USTM), BP 943, Franceville, Gabon; 2 Centre National de Transfusion sanguine (CNTS), BP 13895, Libreville, Gabon; 3 Division of Biostatistics, Mayo Clinic Scottsdale, Scottsdale, AZ USA; 4 Department of Hematology/Oncology, Mayo Clinic Scottsdale, Scottsdale, AZ USA; 5 University of Cambridge, Cambridge, UK

**Keywords:** Syphilis, Seroprevalence, Decline, First-time blood donors, Gabon

## Abstract

**Background:**

Very few studies have been conducted on the seroprevalence of syphilis in Gabon. According to the World Health Organization, the average seroprevalence of syphilis has declined from 5.5 to 1.1% in Central Africa. The aim of this study was to test the hypothesis that syphilis decreased in Gabon between 2004 and 2016 and to identify factors involved in this pattern by testing a large sample of first-time blood donors in the capital Libreville.

**Methods:**

The detection of *Treponema pallidum* was done using a Rapid Plasma Reagin test (RPR) and confirmed by an ELISA test using the Biorad Syphilis Total Antibody EIA II kit or BioMerieux Trepanostika TP recombinant. Assays were performed by dedicated technicians according to manufacturers’ recommendations and following the laboratory standard operating procedures. Test results were manually transferred into the laboratory Excel files and hand-written in the laboratory logbook for syphilis testing. Logistic regression was used to assess the impact of sociodemographic characteristics on syphilis marker seroprevalence in both univariate and multivariable analysis. Odds ratios (OR) and 95% confidence intervals were calculated.

**Results:**

The seroprevalence of syphilis markers was 8.4% (95% CI = 7.9–8.9) in 2004 and 2.4% (95% CI = 2.1–2.7) in 2016. The difference was significant [OR = 3.78; 95% CI (3.26–4.38); *P* < 0.001]. The decrease in syphilis seroprevalence was significant in both women and men and in each age group in univariate analysis. In multivariable analysis, controlling for all sociodemographic factors, the decrease in syphilis seroprevalence from 2004 to 2016 remained significant (OR = 3.29; 95% CI = 2.88–3.88, *P* < 0.001). The seroprevalence of syphilis decreased significantly in men compared to women and young donors compared to donors aged ≥36 years.

**Conclusions:**

This study shows a significant decline in syphilis seroprevalence in first-time blood donors in Libreville, Gabon. Government actions, including multiple HIV prevention activities, are a likely part of this decline.

## Background

Syphilis is essentially a sexually transmitted disease. It can also be transmitted by contact with injured mucosa, by blood transfusion and from pregnant women to their newborn that could have adverse effects on nearly 80% of pregnancies [1].

According to the World Health Organization (WHO), 6 million new syphilitic infections are contracted each year despite the existence of effective preventive and therapeutic measures [2]. In sub-Saharan Africa (SSA), syphilis is widespread in the general population, and its seroprevalence in some countries’ blood donors may reach 25% [3]. However, prevalence is highly dependent on the testing algorithm and test quality [4, 5].

In Gabon, from 1979 to 1982, blood transfusion was provided by a blood bank at the Hospital Center of Libreville. Since 1986, the National Blood Transfusion Center (NBTC) ensures the qualification of blood donations by testing for infectious markers such as human immunodeficiency virus (HIV), hepatitis B and C (HBV and HCV) and the detection of antibodies against *Treponema pallidum pallidum,* the etiological agent of venereal syphilis, using 3rd-generation manual ELISA and recently 4th-generation semi-automated (EVOLIS BioRad) or automated ELISA (Cobas 6000 e601).

Syphilis remains a major public health problem in SSA, even though averaged seroprevalence appears to have decreased in Central Africa from 5.5 to 1%, while in Eastern and Southern Africa it has increased from 0.3 to 0.9% [6]. Very few recent studies have been conducted on the epidemiology of syphilis in Gabon [7, 8]. Previous studies have shown seroprevalence of 13.3% in Franceville adults in 1988 [9]; in 1989, 11.4% in pregnant women [10] and 8% in a semi-rural population [11] and 4.6 and 2.1% in first-time blood donors in 2009 and 2015, respectively [8]. Most of these studies have indicated a downward trend in the seroprevalence of syphilis in Gabon.

The aim of this study was to test the hypothesis that syphilis seroprevalence decreased in Gabon between 2004 and 2016 and to identify factors involved in this pattern by testing a large sample of first-time blood donors in the capital Libreville.

## Methods

### Study site

Gabon is located in Central Africa, straddling the Equator, and has an area of 267,667 km^2^. The bordering countries are Cameroon in the North; Equatorial Guinea in the North-West, Republic of Congo in the East and South and the Atlantic Ocean in the West on 800 km of coast. The country has a population of 1,811,079 inhabitants, with a density of 6.8 inhabitants per km^2^, and a population growth rate of 3.1%. The Gabonese population is mainly young (54.6% under 25 years). At present, 87% of the population lives in urban areas, concentrated on 1.1% of the national territory (“http://www.ga.pnud.org”). The only NBTC is located in the Gabonese capital, Libreville, with a cosmopolitan population of 703,940 inhabitants [12], comprising just over one third of the Gabonese population. In addition to the Libreville standalone NBTC, there are 14 hospital-linked blood banks located in regional hospitals across the country.

### Blood donors

The study was carried out at the NBTC, which oversees all components of the blood donation chain including collection, screening for transfusion transmitted infections (TTIs) and distribution of blood components. Before the computerized record of donors and donations in 2000, few data were available to study blood-borne pathogens among blood donors in Gabon. The most complete and reliable data from Libreville blood centre was from 2004. The majority of the blood was collected at the NBTC in Libreville. A few donations were collected in mobile sessions, mostly in secondary schools but also in universities. In addition, in a previous tri-annual study between 2009 and 2015, we observed a decrease in the seroprevalence of syphilis in blood donors [8]. We therefore selected the years 2004 and 2016 to test the hypothesis that the prevalence of syphilis has decreased in Gabon.

A retrospective analysis of curated data on blood donors collected between 2004 and 2016 was conducted. All apparently healthy voluntary non-remunerated donors (VNRD) and family/replacement donors (FRD) were selected after responding to a range of questions including medical history. Individuals aged 15 to 65 years old weighing ≥50 kg were eligible for blood donations. Donors aged 15 years are eligible for blood donations with the agreement of their parents or guardians. All candidate donors responded to questions aimed to exclude at risk donations including individuals who had jaundice or signs of hepatitis, pregnant women and persons with unsafe sexual behavior during the 6 months prior to blood donation. Venous blood was collected in the blood bags following standard aseptic procedures.

### Serological analysis

Markers of *Treponema pallidum* were detected using Rapid Plasma Reagin test (RPR, Cypress, Diagnostic, Langdorp, Belgium). All reactive samples were confirmed in 2004 with Trepanostika TP recombinant (bioMérieux, France) and in 2016 with syphilis Total antibody EIA II kit (Bio-Rad*,* Marnes-la-Coquette, France). The sensitivity and specificity of Trepanostika TP recombinant were 99.1 and 100%, respectively. Those of syphilis Total antibody EIA II kit were 99.56 and 100%, according to the manufacturers. All samples reactive with both initial and secondary assays were considered positive.

### Statistical analysis

Seroprevalence of syphilis was compared by year, overall and according to demographic characteristics (gender, age group, occupation and donor type). Logistic regression was used to assess the impact of these characteristics on seroprevalence in both univariate and multivariable analysis. Odds ratios (OR) and 95% confidence intervals are presented. Statistical Analysis System software (SAS version 9.4, Cary, NC) was used for analysis. *P* values < 0.05 were considered statistically significant.

## Results

### Sociodemographic characteristics of blood donors

The study included 20,651 first-time donors; 10,904 in 2004 and 9747 in 2016 and were 75.7% men and 24.3% women. The mean blood donor age was 29.7 ± 8.1 years. The majority of donors were family donors (70.6%). Most donors were in the age group 25–35 years (44.4%); donors less than 35 years accounted for 75% of the total. There was a higher percentage of VNRDs in 2014 than in 2016 (38.9% versus 18.7%). Police and armed forces and students accounted for 14.7 and 13.8% of donors, respectively. Sociodemographic data are presented in Table 1.

**Table 1:** Sociodemographic characteristics of first-time blood donor in 2004 and 2016

	Years		
	2004	2016	Total
Number of donors	10,904	9747	20,651
Gender N (%)			
Female	2773 (25.4)	2239 (23.0)	5012 (24.3)
Male	8131 (74.6)	7508 (77.0)	15,639 (75.7)
Age groups N (%)			
<18 years	127 (1.2)	15 (0.2)	142 (0.7)
18–24 years	3114 (28.6)	3042 (31.2)	6156 (29.8)
25–35 years	4463 (40.9)	4713 (48.4)	9176 (44.4)
36–45 years	2404 (22.0)	1525 (15.6)	3929 (19.0)
>45 years	796 (7.3)	452 (4.6)	1248 (6.1)
Blood donor type N (%)			
VNRD	4246 (38.9)	1822 (18.7)	6068 (29.4)
FRD	6658 (61.1)	7925 (81.3)	14,583 (70.6)
Occupation N (%)			
Healthcare workers	58 (0.5)	89 (0.9)	147 (0.7)
Students	298 (2.7)	2545 (26.1)	2843 (13.8)
Teachers/Researchers	98 (0.9)	206 (2.1)	304 (1.5)
Police and armed forces	1846 (16.9)	1196 (12.3)	3042 (14.7)
Drivers	162 (1.5)	613 (6.3)	775 (3.8)
Government employees	234 (2.1)	437 (4.5)	671 (3.2)
Tailors and Hairdressers	84 (0.8)	282 (2.9)	366 (1.8)
Hotel and restaurant workers	12 (0.1)	54 (0.6)	66 (0.3)
Merchants	24 (0.2)	98 (1.0)	122 (0.6)
Others	8088 (74.2)	4227 (43.4)	12,315 (59.6)

*VNRD* volunteer non-remunerated donor, *FRD* Family/Replacement donor

### Decline in the seroprevalence of syphilis among first-time blood donors

Seroprevalence of confirmed syphilis markers was 8.4% (95% CI = 7.9–8.9) in 2004 and 2.4% (95% CI = 2.1–2.7) in 2016. Seroprevalence was nearly four times lower in 2016 compared to 2004 (OR = 3.78; 95%CI = 3.26–4.386; *p* < 0.001). While the overall prevalence of syphilis markers was significantly lower in females than in males in 2004 (*P* < 0.001), the decline observed in 2016 was by a factor of three in females and of four in males and the difference between sexes was no longer significant (*P* = 0.28).

Factors influencing the decline of syphilis seroprevalence in blood donors were examined (Table 2). The observed considerable decline was in part related to demographic factors of the donor population. For instance, in 2016, the proportion of students was 2.7% in 2004 and 26.1% in 2016. Similarly, the proportion of FRD was 61.1% in 2004 and increased to 81.3% in 2016. As a result, the proportion of donors below age 35 years was 9.1% higher in 2016. The dramatic decline of syphilis prevalence in the police and armed forces suggests selection or testing in this professional category. This suggests that part of the observed decrease in syphilis prevalence was related to population change.

**Table 2:** Univariate analysis of syphilis seroprevalence among first-time blood donors according to their sociodemographic characteristics

	Years			
	2004	2016		
Characteristics	% (n/N)	% (n/N)	OR (95% CI)	*P*-values
	8.4 (912/10904)	2.4 (230/9747)	3.78 (3.26–4.38)	<0.001
Gender				
Female	6.3 (175/2773)	2.1 (46/2239)	3.21 (2.31–4.46)	<0.001
Male	10.0 (737/8131)	2.5 (184/7508)	3.97 (3.37–4.68)	<0.001
Age groups (years)				
<18	4.7 (6/127)	0/15	NA	NA
18–24	4.6 (143/3114)	1.0 (30/3042)	4.83 (3.25–7.19)	<0.001
25–35	6.3 (282/4463)	1.7 (80/4713)	3.91 (3.02–5.02)	<0.001
36–45	12.5 (301/2404)	5.0 (76/1525)	2.73 (2.10–3.54)	<0.001
>45	22.6 (180/796)	9.7 (44/452)	2.71 (1.90–3.86)	<0.001
First time donor type				
VNRD	8.1 (347/4246)	2.0 (36/1822)	4.42 (3.12–6.25)	<0.001
FRD	8.5 (565/6658)	2.5 (194/7925)	3.70 (3.13–4.36)	<0.001
Occupation				
Healthcare workers	5.2 (3/58)	3.4 (3/89)	1.56 (0.31–8.03)	0.63
Students	5.4 (16/298)	1.2 (31/2545)	4.60 (2.48–8.52)	<0.001
Teachers/researchers	9.2 (9/98)	7.3 (15/206)	1.29 (0.54–3.06)	0.73
Police and armed forces	9.1 (168/1846)	1.6 (19/1196)	6.20 (3.84–10.03)	<0.001
Drivers	4.9 (8/162)	3.6 (22/613)	1.40 (0.61–3.20)	0.57
Government employees	3.0 (7/234)	2.3 (10/437)	1.32 (0.50–3.51)	0.77
Tailors and hairdressers	0/84	3.6 (10/282)	NA	NA
Hotel and restaurant workers	0 (0/12)	9.3 (5/54)	NA	NA
Merchants	8.3 (2/24)	3.1 (3/98)	2.88 (0.45–18.28)	0.25
Others	8.6 (699/8088)	2.7 (112/4227)	3.48 (2.84–4.26)	<0.001

*VNRD* volunteer non-remunerated donor, *FRD* family/replacement donor, *OR* Odds ratio, *CI* Confidence Interval

In univariate analysis, the decrease in seroprevalence between 2004 and 2016 was observed in both males and females, in all age group categories, and in VNRDs as well as FRDs. Significant decreases were observed in students, police and armed forces and others occupation categories.

In 2004, the seroprevalence of syphilis was below 5% until age 25 years and increased progressively with age, reaching 22.6% above age 45 years. A decline from 4.7% in 2004 to 0% in 2016 among blood donors < 18 years was observed. The reduction in prevalence was by a factor of four and fivefold in donors aged 25–35 years and 18–24 years but by a factor of just over two in older groups (OR = 2.73, *P* < 0.001 and OR = 2.71, P < 0.001) (Table 2).

In 2016 syphilis marker seroprevalence was four times lower than in 2004 for both VNRD and FRD (OR = 4.42, 95% CI = 3.12, 6.25, P < 0.001 and OR = 3.70, 95% CI = 3.13–4.36, P < 0.001) (Table 2). The difference in seroprevalence between donor types was not significant (*p* = 0.57 for 2004 and *p* = 0.23 for 2016).

With regard to socio-occupational groups, the most dramatic decline in seroprevalence of syphilis was observed among students and police and armed forces. The seroprevalence of syphilis was 4.5 and 5.7 times lower in 2016 than in 2004 in these two professional groups, respectively.

A non-significant reduction in the seroprevalence of syphilis was observed in the other socio-professional categories between 2004 and 2016, in part related to small numbers.

There was a 3.2 times decline in seroprevalence of syphilis markers in the large group of ‘other occupations’.

In multivariable analysis (Table 3), controlling for all sociodemographic factors, the decrease in syphilis seroprevalence from 2004 to 2016 remained significant (OR = 3.29; 95% CI = 2.88–3.88, *P* < 0.001). Syphilis seroprevalence decreased significantly in men compared to women and young donors compared to donors aged ≥36 years. For first-time blood donor types and occupation, syphilis seropositivity was not significantly different between VNRD and FRD and when comparing all professional categories, particularly after adjustment for age (Table 3).

**Table 3:** Multivariable analysis of Syphilis seroprevalence

Characteristics	OR (95% CI)	*P*-value
Year		
2004	3.29 (2.88–3.88)	<0.001
2016	Reference	
Gender		
Female	Reference	0.002
Male	1.29 (1.10–1.51)	
Age groups (year)		
<18	Reference	
18–24	0.89 (0.39–2.06)	0.79
25–35	0.28 (0.56–2.93)	0.56
36–45	2.90 (1.27–6.63)	0.01
>45	5.88 (2.55–13.55)	<0.001
First time donor type		
FRD	Reference	
VNRD	0.94 (0.83–1.08)	0.39
Occupation		
Healthcare workers	Reference	
Students	1.08 (0.45–2.61)	0.87
Teachers/researchers	1.94 (0.77–4.94)	0.16
Police and armed forces	1.28 (0.55–2.98)	0.57
Drivers	1.04 (0.42–2.59)	0.93
Government employees	0.62 (0.24–1.63)	0.33
Tailors and hairdressers	0.83 (0.29–2.35)	0.72
Hotel and restaurant workers	3.27 (0.94–11.36)	0.06
Merchants	1.36 (0.40–4.64)	0.62
Others	1.27 (0.55–2.92)	0.57

## Discussion

In this study we showed a significant reduction in the seroprevalence of syphilis among first-time blood donors in Libreville between 2004 and 2016. Syphilis seroprevalence declined from 8.4% in 2004 to 2.4% in 2016. Previous studies in Gabon in the 1980s showed higher seroprevalence of syphilis from 11.4 to 13.3% [9, 10] compared to seroprevalence of 4.6 and 2.1% obtained in the 2000s [8, 13]. Comparing data collected in 2004 and 2016 is a limitation of the study but data from 2005 to 2015 was not available in full and could not be used reliably. It is possible that part of the apparent decline in prevalence might be related to improved assays, or an effect of confirmation that substantially (79%) decreases the initial reactivity rate of the first test, as shown in Ghana [5]. The decline in seroprevalence of syphilis obtained in this study is higher than that observed in studies in Zimbabwe and Morocco among pregnant women attending antenatal clinics [14] and in Ethiopia where a decline from 2.4 to 0.6% was observed [15].

Syphilis seroprevalence regression of 3.8 to 2% between 2002 and 2011 and 9.8 to 2.8% between 1994 and 2011 were observed in pregnant women tested with RPR confirmed by TPHA in Rwanda and Zambia, respectively [16, 17].

The decline we observed is consistent with WHO data showing a fall from 5.5 to 1.1% in the average seroprevalence of syphilis in Central Africa [6]. Decline in seroprevalence of syphilis between 2004 and 2016 may be associated with reduction of sexually transmitted infections (STIs). Indeed, the dramatic fall in the HIV pandemic in Gabon may have been accompanied by a decline in syphilis seroprevalence [18]. From 2008 to 2012, condom use in Gabon has increased among men (76.5%) and women (55%) aged 15–24 years [19]. This use has been effective in preventing HIV and syphilis transmission. In some previous studies in sub-Saharan Africa, the decline of syphilis has been associated with the promotion of safer sex among high-risk groups, the improvement of STI treatment programs through the use of the syndromic approach, systematic screening, self-screening of spouses of positive screened women [20, 21]. The decline of syphilis seroprevalence in our study may also be affected by the urban origin of the majority of blood donors where intensive campaigns against STIs take place; diagnostic tools and quality treatment are available in well-equipped hospital facilities. Indeed, studies have shown a preponderance of syphilitic infection in rural areas because of the low level of education of the populations, limited access to health care and a higher level of poverty [22, 23].

The seroprevalence of syphilis decreased significantly in young blood donors between 2004 and 2016. It was higher among older blood donors, agreeing with the results of an earlier study in Gabon [11]. Similar results were obtained in Cameroon by Ankouane et al., [24] and in other studies in Iran [25] and Israel [26]. The regression of syphilis could be explained by its low prevalence among predominantly young blood donors (less than 35 years). The seroprevalence of syphilis increased with age in both 2004 and 2016, as previously shown [27]. The highest seroprevalence observed in the oldest subjects could be explained by a longer exposure to infection risk; infections may also be the result of past but healed infections (immunological scars). Some studies, however, have reported a high prevalence of syphilis in young people aged 15 to 28 years [16, 28, 29]. The decline in seroprevalence of syphilis was similar in both VNRDs and FRDs and among all socio-professional categories. However, two previous studies in Zambia and the Democratic Republic of Congo have associated women’s education level with low seroprevalence of syphilis [17, 23]. An earlier study in Gabon reported a seroprevalence of syphilis of 0.73% in a student population in Libreville [30].

## Conclusions

This study has shown that the seroprevalence of syphilis has decreased in the population of first-time blood donors, which may be a reflection of the adult general population. However, we have no evidence of how representative of the general population the population of first time blood donors might be. Government actions, including multiple HIV prevention activities, are a likely part of this decline.

## Abbreviations

CI: Confidence interval
FRD: Family/replacement donor
NBTC: National blood transfusion center
OR: Odd ratio
RPR: Rapid Plasma Reagin test
SSA: Sub-Saharan Africa
WHO: World health organization
